# Afferent Loop Syndrome after Roux-en-Y Total Gastrectomy Caused by Volvulus of the Roux-Limb

**DOI:** 10.1155/2016/4930354

**Published:** 2016-06-26

**Authors:** Hideki Katagiri, Kana Tahara, Kentaro Yoshikawa, Alan Kawarai Lefor, Tadao Kubota, Ken Mizokami

**Affiliations:** ^1^Department of Surgery, Tokyo Bay Urayasu Ichikawa Medical Center, 3-4-32 Todaijima, Urayasu, Chiba 279-0001, Japan; ^2^Department of Surgery, Jichi Medical University, 1-3311 Yakushiji, Shimotsuke, Tochigi Prefecture 329-0498, Japan

## Abstract

Afferent loop syndrome is a rare complication of gastric surgery. An obstruction of the afferent limb can present in various ways. A 73-year-old man presented with one day of persistent abdominal pain, gradually radiating to the back. He had a history of total gastrectomy with a Roux-en-Y reconstruction. Abdominal computed tomography scan revealed dilation of the duodenum and small intestine in the left upper quadrant. Exploratory laparotomy showed volvulus of the biliopancreatic limb that caused afferent loop syndrome. In this patient, the 50 cm long limb was the cause of volvulus. It is important to fashion a Roux-limb of appropriate length to prevent this complication.

## 1. Introduction

Afferent loop syndrome is a rare complication of gastric surgery. In general, afferent loop syndrome develops after distal gastrectomy following a Billroth II reconstruction. However, the same condition can occur after a Roux-en-Y reconstruction by stenosis or obstruction of “biliopancreatic limb” [1–3]. We present a patient who developed afferent loop syndrome after total gastrectomy with a Roux-en-Y reconstruction caused by volvulus of the biliopancreatic limb.

## 2. Case Report

A 73-year-old man presented with periumbilical abdominal pain. One day prior to admission, he noticed the gradual onset of abdominal pain. The pain was not severe and he did not seek medical attention at that time. He did not have nausea or vomiting. He was able to eat but tolerated a smaller amount than usual. However, the pain persisted and gradually worsened, and he presented to the hospital.

**Figure 1:**
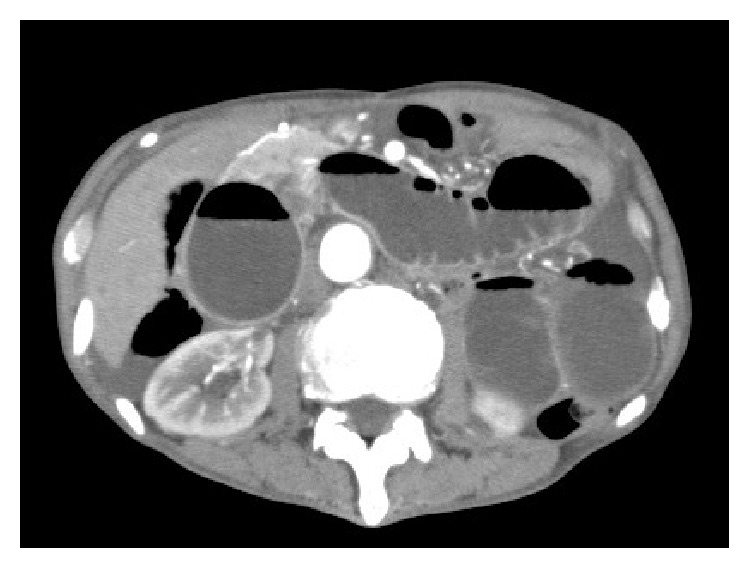
Abdominal computed tomography scan revealed dilation of the duodenum and small intestine in the left upper quadrant.

**Figure 2:**
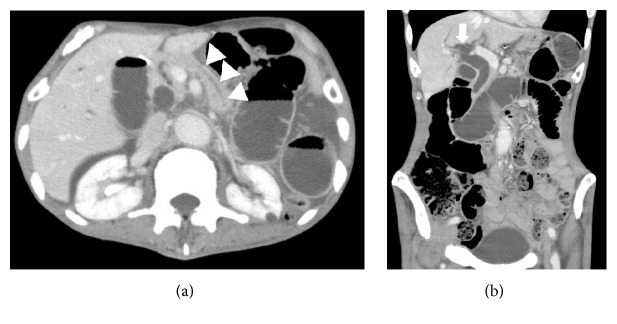
Computed tomography scan images with axial and coronal views showing slight dilation of the main pancreatic duct (arrow heads) and the intrahepatic bile duct (arrow).

The patient has a past medical history of hyperthyroidism, aortic valve replacement due to aortic insufficiency one month prior to presentation, and total gastrectomy for gastric cancer two years previously. He has had two episodes of adhesive small bowel obstruction, one of which required operative intervention. On admission, his vital signs were within normal limits except for a respiratory rate of 30/min. Physical examination showed tenderness from the left flank to the right upper quadrant with palpable loops of dilated intestine. During the physical examination, he started complaining of back pain. Laboratory data showed slight elevation of lipase and alkaline phosphatase. Abdominal computed tomography scan with intravenous contrast was obtained, which showed dilated duodenum and small intestine in the left upper quadrant (Figure 1). In addition, slight dilation of the main pancreatic duct and the intrahepatic bile duct was seen (Figures 2(a) and 2(b)).

**Figure 3:**
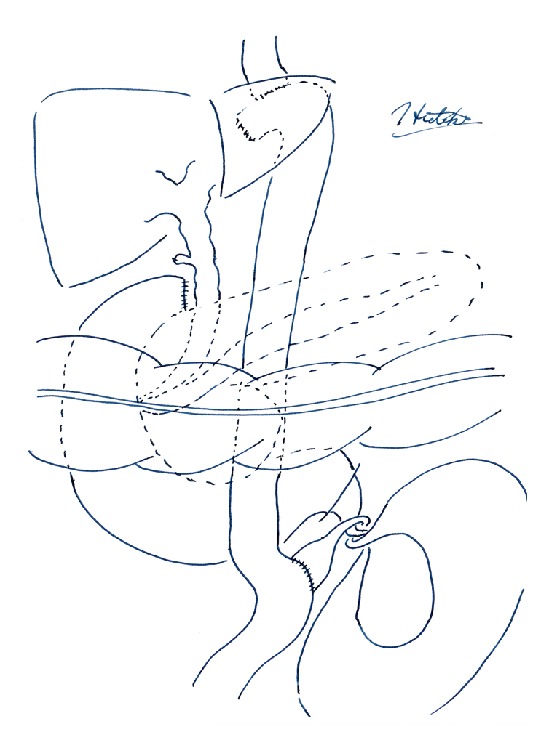
Schematic diagram of intraoperative findings. The patient previously underwent total gastrectomy and cholecystectomy. The afferent (biliopancreatic) limb was twisted 360 degrees.

Based on these findings, afferent loop syndrome was highly suspected, and we performed exploratory laparotomy urgently. On exploration, there were dilated loops of small intestine without adhesions in the left upper quadrant. The jejunum, from the ligament of Treitz to the site of the jejunojejunal anastomosis, was twisted 360 degrees counterclockwise (Figure 3). We reduced the volvulus manually without difficulty and the dilation of the bowel rapidly resolved. There was no evidence of intestinal necrosis. The postoperative course was uneventful and the patient was discharged from hospital.

## 3. Discussion

Afferent loop syndrome is a rare complication that occurs in 0.2 to 1.0% of patients after gastrectomy with a Billroth II or Roux-en-Y reconstruction [1, 2, 4, 5]. To be accurate, the afferent limb is not only the afferent limb in patients following a Billroth II reconstruction but also refers to the biliopancreatic limb in patients following a Roux-en-Y reconstruction, in this discussion. Afferent loop syndrome can be caused by internal herniation, kinking at the anastomotic site, adhesions, stomal stenosis, a gastrointestinal stone, recurrent malignancy, and volvulus [1–5]. An obstruction of the afferent limb disrupts the flow of bile and pancreatic juice, resulting in acute pancreatitis or obstructive jaundice [4]. In some patients, afferent loop syndrome can rapidly develop, followed by perforation or peritonitis [1]. In the present patient, the volvulus resulted in complete obstruction of the afferent limb and the patient's condition worsened rapidly.

The diagnosis of afferent loop syndrome is challenging because the symptoms are generally nonspecific [1]. Abdominal pain is one of the common symptoms. Vomiting may occur in patients with afferent loop syndrome; however, it is very difficult to assess. In patients with incomplete obstruction, patients may vomit and the vomitus can contain bile. However, in patients with complete obstruction, patients do not vomit (as in the present patient), because the afferent limb is completely obstructed. As the condition worsens, patients may develop acute pancreatitis or obstructive jaundice, which also makes establishing the diagnosis challenging. Early diagnosis and intervention is important to decrease the mortality rate, especially when the condition develops acutely [1]. In the present patient, slight elevation of lipase and alkaline phosphatase, slight dilation of the common pancreatic duct and the bile duct, and the onset of back pain all suggested high intraluminal pressure and reflux of intestinal fluid to the ducts. Prolonged high intraluminal pressure can result in intestinal necrosis, making early diagnosis and intervention essential.

In the present patient, volvulus of the afferent limb caused afferent loop syndrome. In general, the length of jejunum between the ligament of Treitz and the jejunojejunal anastomosis is typically 20 to 30 cm. However, in the present patient, it was longer than 50 cm. This is longer than usual and may have facilitated the development of volvulus. This patient emphasizes the importance of the length of the afferent limb in gastric surgery. As it is easy to shorten the afferent limb, especially for a Roux-en-Y reconstruction, fashioning an appropriate length for the afferent limb can potentially prevent this complication.

Treatment of afferent loop syndrome depends on the etiology. In the present patient, reduction of the volvulus resolved the condition. In patients with benign etiologies, surgical management including adhesiolysis, bypass, or reconstruction of the limb can generally resolve the cause [2]. In patients with afferent loop syndrome caused by recurrent tumor, the goal of the treatment changes to palliation. In some settings, drainage by percutaneous or endoscopic stent placement has been reported to achieve palliation in patients with cancer [4, 6].

In conclusion, afferent loop syndrome is a rare complication after gastric surgery. Although rare, it is important for surgeons to fashion an afferent limb of appropriate length to prevent this complication.
